# Cytotoxic response persists in subjects treated for tuberculosis decades ago

**DOI:** 10.1186/1471-2334-13-573

**Published:** 2013-12-05

**Authors:** Laura E Savolainen, Pekka Koskivirta, Anu Kantele, Heikki Valleala, Liana Pusa, Riitta Tuompo, Benita Westerlund-Wikström, Tamara Tuuminen

**Affiliations:** 1 Department of Bacteriology and Immunology, Haartman Institute, University of Helsinki, PL 21, Helsinki 00014, Finland; 2 Division of Infectious Diseases, Department of Medicine, Helsinki University Central Hospital, Helsinki, Finland; 3 Institute of Clinical Medicine, Department of Medicine, University of Helsinki, Helsinki, Finland; 4 Division of Rheumatology, Department of Medicine, Helsinki University Central Hospital, Helsinki, Finland; 5 Länsi-Uusimaa Hospital, Tammisaari, Finland; 6 Division of General Microbiology, Department of Biosciences, University of Helsinki, Helsinki, Finland; 7 Eastern Finland Laboratory Centre Joint Authority Enterprise, Mikkeli, Finland

**Keywords:** Tuberculosis, CTLs, Immunodiagnostics, Granzyme B, CD107a, Perforin, Memory T cells

## Abstract

**Background:**

Data exploring the potential use of effector molecules produced by cytotoxic T lymphocytes (CTLs) in the immunodiagnostics of tuberculosis (TB) are scarce. The present study focused *a)* to gain an insight into the discriminatory power of CTLs in patients with acute pulmonary or extra-pulmonary TB, or latent tuberculosis infection (LTBI); and *b)* to evaluate the influence of various anti-TB therapeutic schemes on the immunological profiles of residual CTLs.

**Methods:**

Immunological signatures of antigen-specific CTLs were explored in patients with active pulmonary and extra-pulmonary TB, LTBI and in those treated for TB decades ago by using ELISPOT, intracellular flow cytometry and extracellular CD107a detection.

**Results:**

No difference was seen between active TB, LTBI or any of those treated for TB in the ELISPOT analysis of antigen-specific Granzyme B (GrB), Perforin (Prf) and interferon-gamma (IFN-γ) producing lymphocytes, the FACS analysis of the intracellular expression of IFN-γ, or the surface expression of CD107a degranulation factor of both CD8^+^ and CD4^+^ antigen-specific T cell subsets. The effector memory (T_EM_) phenotype proved predominant in the surface marker profiling both in active TB and LTBI. The proportion of the CD107a degranulation factor proved higher in the central memory (T_CM_) than in the other cell subsets in all the study groups. Interestingly, functionally and phenotypically similar CTLs profiles were observed in active TB, LTBI and in all the three groups treated for TB.

**Conclusion:**

The phenotypic and functional profiling of CTLs has a limited potential in the immunodiagnostics of active TB. Antigen-specific CTLs persist in patients treated for TB decades ago regardless of the efficacy of implemented and completed anti-TB therapy.

## Background

The interferon-gamma (IFN-γ) releasing antigen-specific T cells have been extensively exploited in the immunodiagnostics of tuberculosis (TB) [1,2]. However, it has become obvious that these IFN-γ release assays (IGRA) lack diagnostic potential to discriminate active TB from latent tuberculosis infection (LTBI) or the latter from immunologic memory [3,4]. Therefore, new biomarkers or biosignatures of the host response to *Mycobacterium tuberculosis (Mtb)* invasion are urgently needed. Ideally, these biomarkers should provide correlates of both cure and risks for disease progression [5,6]. The polyfunctionality of antigen-specific T cells has recently been investigated, but the results appear partially contradictory [7,8]. In active TB, T lymphocytes particularly of the CD4^+^ T cell subset have been reported to simultaneously express multiple effector molecules, e.g. IFN-γ, TNFα and surface degranulation markers CD107a as well as b, but not IL-2 [7]. Day et al. 2011 have shown that the decline of the bacterial load during anti-TB treatment is accompanied by an increase in the frequency of polyfunctional IFN-γ^+^IL-2^+^TNF-α^+^ cells. The same has also been reported for CD8^+^ T cells [8]. Disappointingly, however, in a recent large prospective evaluation, T cells producing a single cytokine as well as polyfunctional T cells had only a limited role in the diagnostics of active TB [9]. Instead, antigen-specific memory CD4^+^ T cells expressing programmed death-1 receptor or CD27 were suggested as a tool for differentiating individuals with LTBI from those having received adequate anti-TB treatment [10].

Cytotoxic T lymphocytes (CTLs) and natural killer cells (NK) destroy transformed tumor cells and cells infected with intracellular pathogens, such as viruses or *Mtb *[11,12]. CTLs and NK cells have similar effector functions, but are triggered by distinct receptors [13]. The elimination of the target cell is mediated by two major pathways: ligand-ligand cell death or release of cytolytic molecules, e.g. Granzyme B (GrB) and Perforin (Prf) from intra-cytoplasmic granules [11,12]. These cytolytic effector molecules are preformed within cytoplasmic granules. Spontaneous leakage of these molecules is prohibited by a lipid bilayer containing lysosomal-associated membrane glycoproteins LAMP1 (CD107a), LAMP2 (CD107b), and LAMP3 [14]. Once a cognate antigen is recognized by T-cell receptors (TCR), CTLs become activated, which lead to the polarization of microtubules [12]. This in part facilitates the migration of lytic microgranules towards immunological synapses formed between the CTL and the target cell. As the granules reach the cell surface, the content of the granule with GrB and Prf is released. Simultaneously, the membrane proteins CD107a and CD107b become surface-exposed, thus providing a marker of degranulation and cytotoxic killing [15]. Until now, the cytotoxic signatures have not yet been fully exploited in TB diagnostics because of the lack of reliable and robust techniques in cytotoxic studies [16]. Although the methods measuring the functionality of CTLs, e.g. GrB and Prf ELISPOTs, and extracellular CD107a detection have been evaluated [15-17], these methods are not widely used as yet.

We have previously investigated the immunophenotypic profiles of CD4^+^ T cells in elderly Finnish volunteers who had completed anti-TB chemotherapy decades ago [4]. The residual responses to *Mtb* specific antigens proved highly variable. The CD4^+^ T cell subsets carried an immunophenotype compatible with effector memory T_EM_ (CD4^+^CCR7^-^CD28^-^CD45RO^+^CD45RA^-^) cells. This observation left us with a critical question: What makes the host continue to produce T-cell clones with effectory functions even after successful chemotherapy? Is it because of the antigen or bacterial persistence?

This research was conducted to gain an insight into the discriminatory power of CTLs in TB immunodiagnostics. More specifically, we compared the frequencies, the functional and the phenotypic profiles of CTLs in patients with acute pulmonary or extra-pulmonary TB or LTBI, and in healthy BCG vaccinees. The second task was to investigate whether various anti-TB therapeutic schemes would influence the immunological profiles of residual CTLs and to evaluate whether these findings would correlate with our previously published data on antigen-specific CD4^+^ T cells in the same study groups [4].

## Methods

### Patient groups and clinical samples

The study comprised 6 study groups: 21 patients with active TB and 11 patients with a positive IGRA result, who had a history of TB exposure, but no symptoms of active disease, and therefore by definition being assigned to the group of LTBI. In addition, cryopreserved samples from patients with TB diagnosed and treated decades ago were retrieved from the freezer. The samples have been described in details earlier [4]. In brief, the samples were grouped as follows: 6 participants were treated with surgery only, and did not receive any anti-TB treatment (Surg.); 23 had received partial anti-TB treatment before the rifampicin (RMP) era (Part.); and 8 were treated with RMP as a part of modern three-drug therapy (Mod.). In addition, we tested six samples from healthy Bacillus Calmette-Guérin (BCG) vaccinees in which LTBI was excluded by a negative IGRA test. All enrolled persons have been living in a low TB prevalence country, thus the re-exposure to *Mtb* is highly unlikely. The demographic and clinical data of samples enrolled in this study are presented in Table 1.

**Table 1:** Demographic and clinical data

**Patient groups**	**n**	**Finns n (%)**	**Age range**	**Female sex n (%)**	**AFB pos. n (%)**	**Culture pos. n (%)**	**Other method n (%)**
Active-TB (TB)							
Pulmonary	18	14 (78)	27 - 81	6 (33)	14 (78)	17 (94)	Clinical presentation and response to treatment: 1 (6)
Other organs	3	2 (67)	33 - 62	1 (33)	0 (0)	3 (100)	
LTBI	11	10 (91)	43 - 80	4 (36)	n/d	n/d	IGRA pos: 11 (100)
Treated patients - no chemotherapy (Surg.)	6	6 (100)	53 - 83	2 (33)	n/d	n/d	n/d
Treated patients - partial treatment (Part.)	23	23 (100)	71 - 90	18 (78)	n/d	n/d	n/d
Treated patients - modern three-drug treatment (Mod.)	8	7 (86)	45 - 84	7 (86)	n/d	n/d	n/d
Healthy BCG-vaccinees (BCG)	6	6 (100)	33 - 44	6 (100)	n/d	n/d	IGRA neg.: 6 (100)

n/d = not defined.

All but one patient in the group with active TB had positive *Mtb* culture, or acid fast bacilli at staining, or both. One patient was diagnosed on the basis of clinical and radiological findings and a good response to anti-TB treatment. The samples were taken no later than 2 weeks after the diagnosis. From the group with active TB, 18 had pulmonary presentation, 2 had TB of mesentery lymphatic nodes and 1 had TB spondylitis.

### Recombinant proteins

Recombinant *Mtb* proteins were expressed in and purified from *Escherichia coli* M15. Genes encoding culture filtrate protein-10 *(cfp-10)* and early secretory antigen target-6 (*esat-6)* were PCR- amplified using purified chromosomal *Mtb* DNA as the template and primers designed to amplify the coding sequence. The genes were cloned into the pQE30 vector using the QIAexpress System (QIAGEN, GmbH, Germany). The recombinant proteins containing an N-terminal histidine tag (rCFP-10 and rESAT-6) were overexpressed and affinity purified, as previously described [18]. The purity and the apparent molecular weight of the expressed proteins were confirmed by SDS-PAGE gel electrophoresis and Western blotting with anti-His antibodies (Clontech Laboratories, CA, USA). Concentration of endotoxin in the protein samples was determined by *Limulus amebocyte* lysate assay (GenScript inc., Piscataway, NY, USA), and was found to be below 0.1 EU/ml.

### ELISPOT-assays

Ficol density gradient (Amersham Biosciences AB, Uppsala, Sweden) was used to isolate peripheral blood mononuclear cells (PBMCs). To avoid the stimulation of cells with fetal calf serum (FCS), the cells were preserved in CryoABC Kit (CTL-Europe, GmpH, Bonn, Germany) in liquid nitrogen until use [19]. The GrB, Prf and IFN-γ ELISPOTs were performed with the commercial kits according to manufacturer’s instructions (Mabtech, Nacka Strand, Sweden). In GrB and Prf assays the cells were stimulated immediately after thawing for 22 hrs with purified protein derivative (PPD) (Statens Serum Institut (SSI), Copenhagen, Denmark), rCFP-10 and rESAT-6 (10 μg/ml, each). In the IFN-γ assay, the stimulation was carried out with PPD (10 μg/ml), and CFP-10 and ESAT-6 peptide pools, 50 μl/100 μl cell suspension (Oxford Immunotec, Oxford, UK). In all assays 2×10^5^ PBMCs were plated per well, and phytohemagglutinin (PHA) (Oxford Immunotec) stimulation was performed to assure the viability of cells. The spots were counted and their sizes were analyzed with the ELISPOT-reader (BIO-SYS-GmbH, Karben, Gemany). Media controls were subtracted to calculate the net values. In a few patients, the number of cells isolated proved too low to allow all experiments to be carried out.

### Flow cytometric staining and analysis

PBMCs purified with density gradient and stored in liquid nitrogen were thawed, and 5×10^5^ cells were incubated with PPD (10 μg/ml), or with media alone, overnight at 37°C with 5% CO_2_ in RPMI-10% FCS (HaartBio, Helsinki, Finland; Sigma-Aldrich, Saint Louis, USA). FITC-conjugated CD107a antibody (5 μl/1×10^6^ cells, eBioscience, Inc., San Diego, CA, USA) was added to the cells before stimulation. After 2 hours incubation, Brefeldin A (eBioscience) was added. Then, after an overnight incubation, the cells were washed with PBS-0,1% FCS, and surface-stained in the same buffer with anti-CD8-Horizon-V450 (5 μl/1×10^6^ cells) and anti-CD4-Alexa Fluor® 700 (0.4 μg/1×10^6^ cells) (Becton Dickinson (BD), Franklin Lakes, NJ, USA) 30 min on ice. After treatment with a fixation buffer for 20 min in RT (eBiosciences), the cells were washed two times with permeabilzation buffer (eBiosciences). The cells were stained for 20 min in RT with anti-IFN-γ-PE-Cy7 (0.2 μg/1×10^6^ cells) (eBioscience) in a permeabilization buffer. After washing with the same buffer the cells were resuspended with PBS-0,1% FCS, and analyzed. The percentages of CD107a and IFN-γ positive cells were calculated. CD4^+^ and CD8^+^ T cells were analyzed separately and the background (unstimulated cells) was subtracted.

For phenotypic characterization, the PBMCs were stimulated and stained for detection of CD107a as described above. After overnight incubation, the cells were washed with PBS-0,1% FCS and surface-stained with anti-CD8-Horizon-V450 (5 μl/1×10^6^ cells) (BD), anti-CD45RA-PE-Cy7 (5 μl/1×10^6^ cells) (eBioscience), and anti-CCR7-PE (5 μl/1×10^6^ cells) (eBioscience). The CD8^+^ T cells with different combinations of CD45RA and CCR7 markers were gated, and CD107a positive cells were analyzed from all of the gates, and the background was subtracted.

### Analysis of flow cytometric results

The samples were analyzed by FACSAria (BD) flow cytometer. To optimize the compensation settings, the BD™ CompBeads Compensation Particles were used (BD). The lymphocytes were gated according to forward and side scatter, and at least 100,000 events were acquired. To analyze the plots, the FACSDiva Version 6.1.3 software (BD) was used.

### Statistics

The data analysis was carried out with GraphPad Prism version 4.0 (GraphPad Software, Inc. San Diego, CA.). ELISPOT and FACS results were analyzed with Kruskal-Wallis test, with Dunn’s multiple comparison, or Mann-Whitney U-test. The correlation analysis was performed with the non-parametric Spearman’s rank correlation test. The *p* value < 0.05 was considered significant.

### Ethics statement

The study was approved by the Southwest Finland district Ethical Committee (DroNo 47/180/2009), Helsinki and Uusimaa Hospital district Ethical Committee of Medicine (DroNo 356/E5/07), Helsinki and Uusimaa Hospital district (149/2010) and University hospital of Kuopio (105/2010). Those patients who were in a regular ward gave written consents. The patients who were in the isolation rooms gave their verbal consents by phone. The day and time of the phone call were documented. All patients received written and verbal information. This was a routine ethical procedure accepted in our hospital.

## Results

### Enumeration of GrB, Prf and IFN-γ-producing T cells

**Figure 1:**
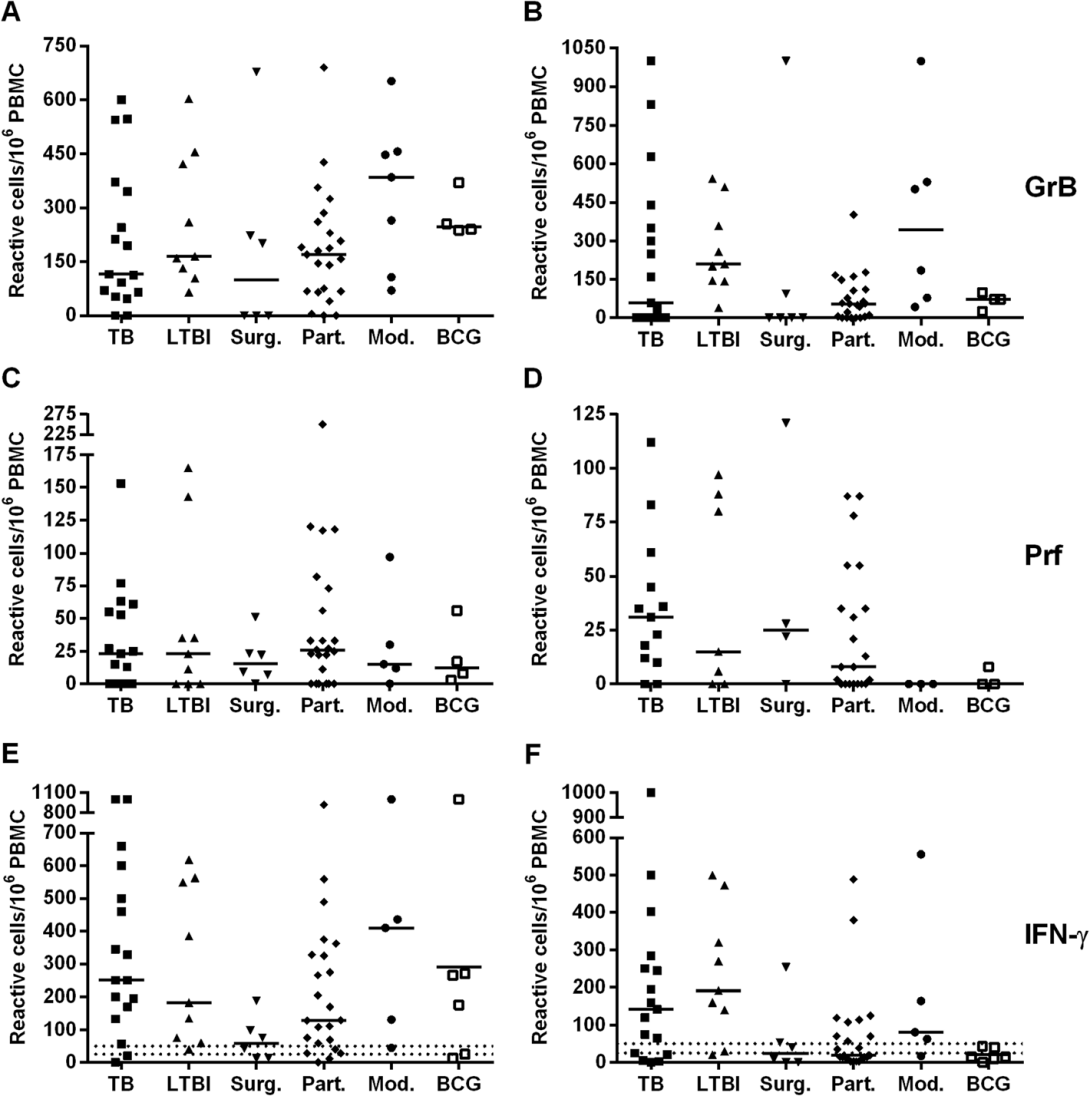
**Frequencies of cells producing effector molecules measured by ELISPOT.** GrB **(A-B)**, Prf **(C-D)** and IFN-γ- producing cells **(E-F)** were stimulated with PPD **(A,C,E)**, rCFP-10 **(B,D)**, and the CFP-10 peptide pool **(F)**. Horizontal bars indicate the medians of the responses. Upper dashed lines represent the cut-offs for the positive IFN-γ results, and lower dashed lines cut-offs for the negative results **(E-F)**. TB, n = 17 (rCFP-Prf n = 13), LTBI, n = 9 (rCFP-Prf n = 7), Surg., n = 6 (rCFP-Prf n = 4), Part., 23 (rCFP-Prf n = 21), Mod., n = 7 (rCFP-GrB n = 6), (CFP-peptides-IFN-γ, PPD-Prf, PPD-IFN-γ n = 5), (rCFP-perforin n = 3), BCG+, n = 6 (PPD-GrB, rCFP-GrB, PPD-Prf n = 4, rCFP-Prf n = 3).

**Figure 2:**
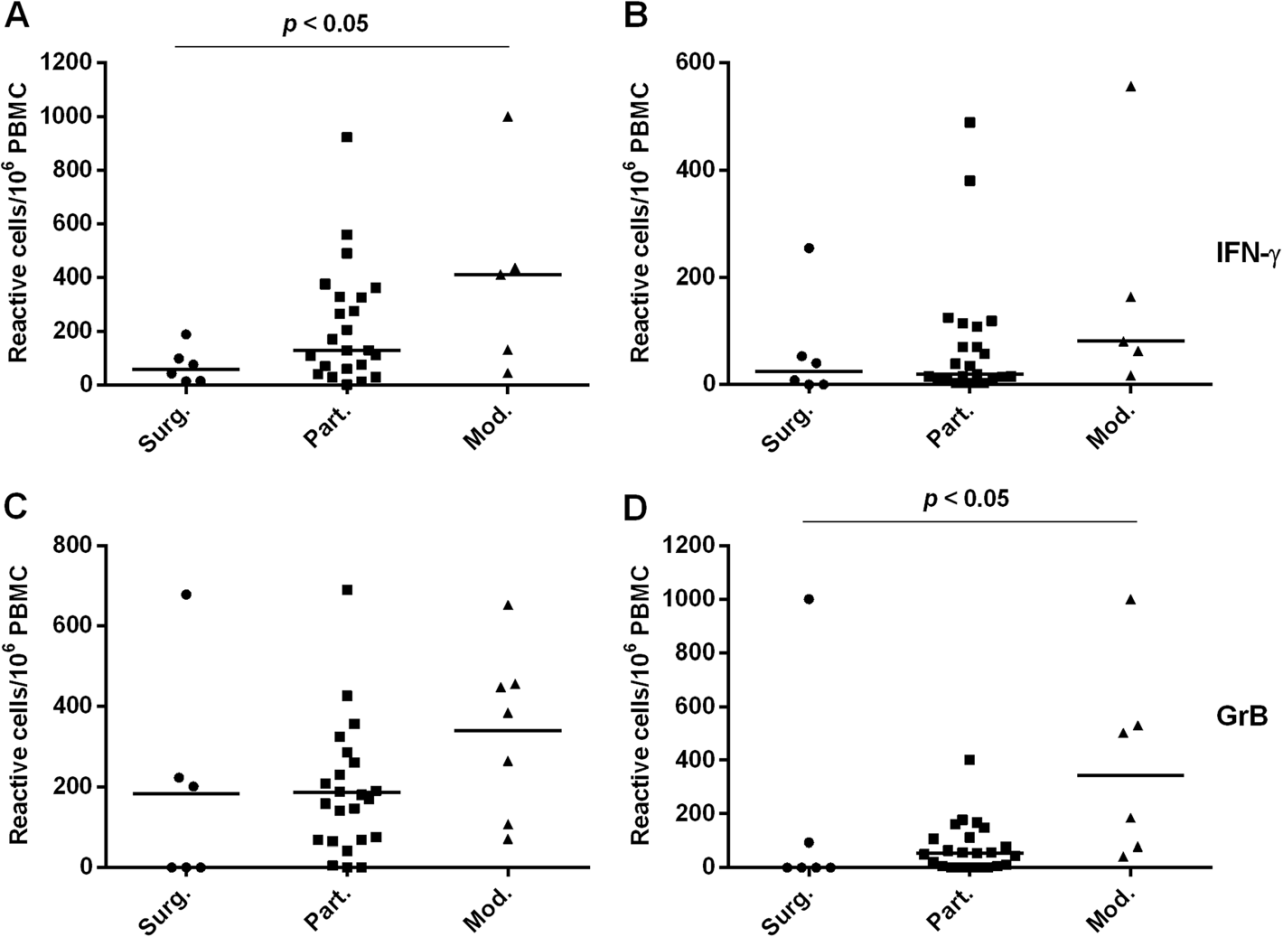
**Comparison of the ELISPOT immune responses in treated patients.** Frequencies of IFN-γ and GrB producing cells when stimulated with PPD **(A,C)**, CFP-10 peptide pool **(B)** or rCFP-10 **(D)**. The median values are shown with horizontal bars. Surg., n = 6, Part., n = 23, Mod., n = 7 (rCFP-GrB n = 6; PPD-IFN-γ, CFP-peptides-IFN-γ n = 5).

To determine the cytolytic potential of the CTLs, the frequencies of antigen-specific GrB, Prf and IFN-γ producing lymphocytes were analyzed with ELISPOT (Figure 1). Inter-individual variations proved high, yet no statistically significant differences were observed between the TB and LTBI groups. In the majority of the study groups, the median frequency for GrB-producing cells was higher than for those producing Prf. Stimulation with rESAT-6 and rCFP-10 induced similar output responses, yet the background was slightly higher with rESAT-6 (data not shown). Noteworthy, the background was high for both markers probably due to handling of cells which resulted in a spontaneous release of effector molecules. Although the groups were small, the frequencies of IFN-γ and GrB producing CTLs proved to differ significantly (p < 0.05) between the subjects receiving the 3-drug therapy (Mod.) and those treated with surgery (Surg.) (Figure 2).

### Flow cytometric studies of CD107a and IFN-γ expression

**Figure 3:**
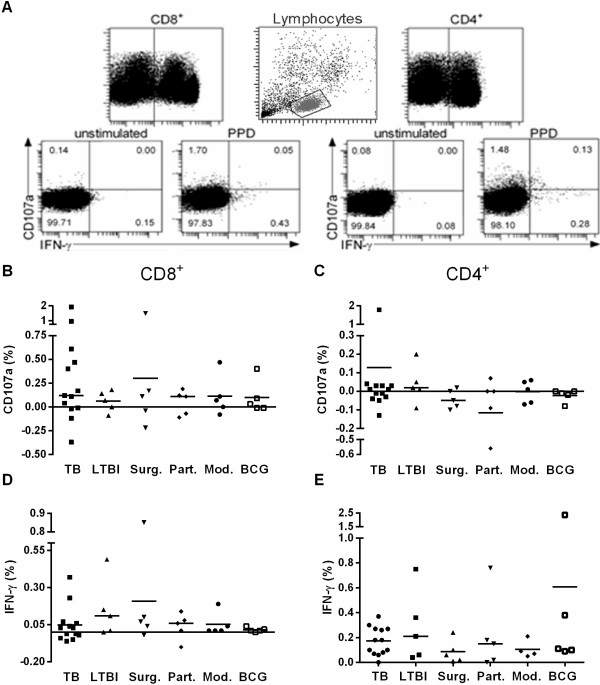
**Flow cytometric analysis for CD107a degranulation marker and IFN-γ production in CD8**^**+ **^**and CD4**^**+ **^**T cell subsets.** The frequencies of CD107a and IFN-γ expressing cells were analyzed from CD8^+^ and CD4^+^ T cell subsets **(A)**. The cells were stimulated with PPD and the percentages of CD8^+^ and CD4^+^ T cells expressing CD107a **(B,C)** and IFN-γ **(D,E)** are calculated after the subtraction of the corresponding results from non-stimulated cells. The horizontal bars represent the medians of the responses. TB, n = 13, LTBI, n = 5, Surg., n = 5, Part., n = 5, Mod., n = 5 (CD4^+^IFN-γ^+^ n = 4), BCG+, n = 5.

The intracellular expression of IFN-γ, and surface expression of CD107a were analyzed by FACS. Gating strategy and representative dot plots are shown in Figure 3A. No statistically significant differences between the study groups were observed. When results from all the subjects were pooled, the frequency of antigen-specific CD107a degranulation factor proved significantly higher (p < 0.01) in the CD8^+^ (Figure 3B) than in the CD4^+^ T cell subset (Figure 3C), whereas reciprocally, a higher production of IFN-γ was found in the CD4^+^ (p < 0.0001) than the CD8^+^ T cell subset (Figure 3D and E). The CD107a and IFN-γ double positive cells yielded very low frequencies in both cell phenotypes (data not shown). In one person treated with modern therapy, the frequency of the CD4^+^ IFN-γ-producing T cells reached 6.74% (data not shown). Taken together, functionally active CTLs were detected in all the study groups.

### Correlation between expressions and production of different markers of cytotoxicity

The correlation between the CD107a expression (FACS) and the production of GrB and Prf (ELISPOT) was studied with the Spearman’s rank correlation test. A positive correlation between GrB and Prf was found only for the LTBI patient group (*r* = 0.78, *p* < 0.05). A significant positive correlation (*r* = 0.41, *p* < 0.05) was detected also between CD8^+^CD107a^+^ expression and GrB production when all the patient samples were subjected to the analysis. Poor correlation between the results from different methods suggests either that the expression of cytotoxicity signature markers occurs independently, or that the markers are short-lived.

### Phenotypic analysis of CD8^+^CD107a^+^ T cells

**Figure 4:**
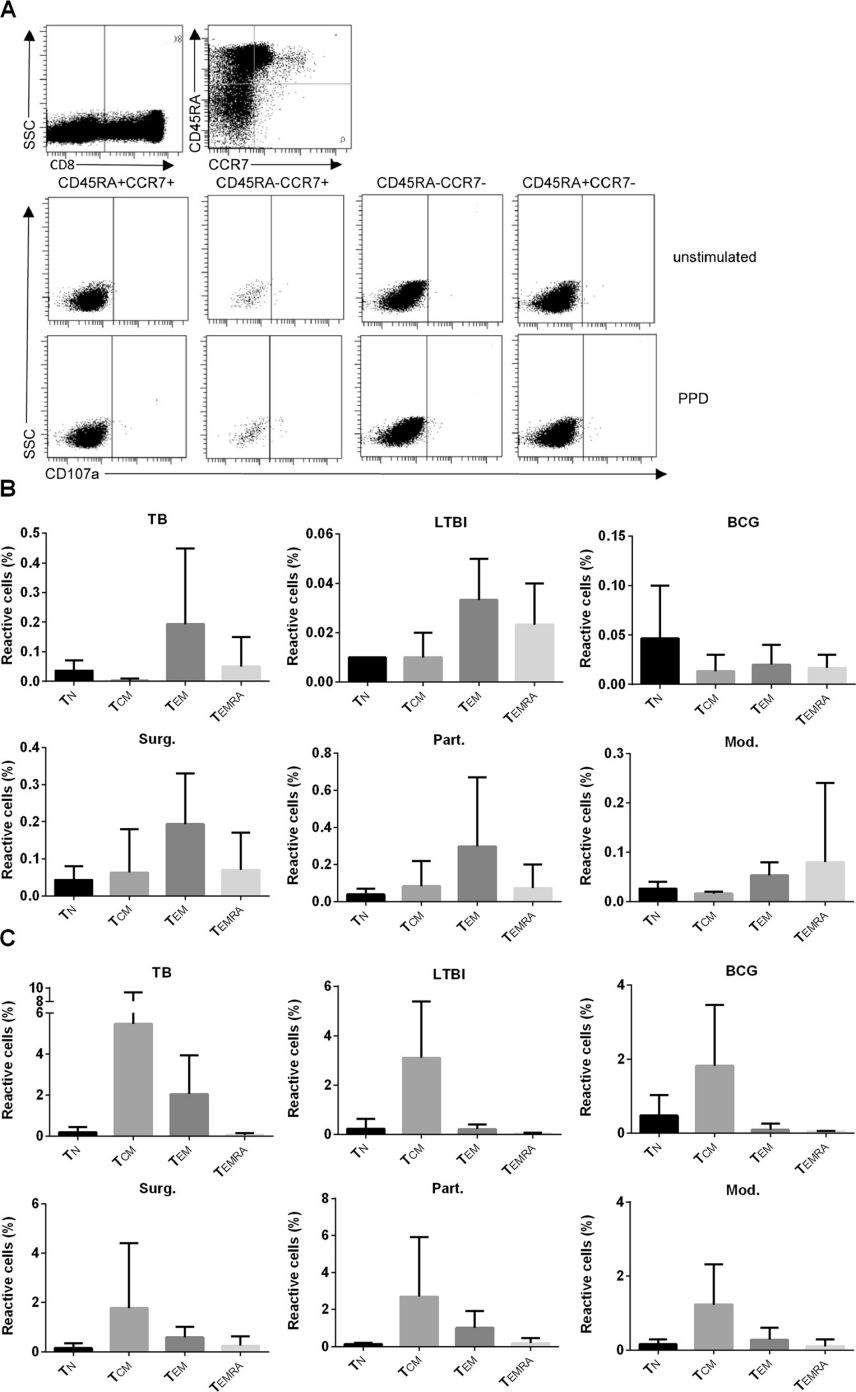
**Phenotypic assessment of the CD8**^**+**^**CD107a**^**+ **^**T cell subsets using the FACS analysis.** The quantities of CD107a expressing cells were analyzed from the CD8^+^ T cell subset with different combinations of CD45RA and CCR7 surface markers after the PPD stimulation **(A)**. The phenotypic distribution of T_N_, T_CM_, T_EM_ and T_EMRA_ cell subsets within the CD8^+^CD107a^+^ T cell pool was assessed in different study groups. The T_EM_ phenotype predominated in the TB., LTBI., Surg. and Part. groups whereas in the BCG vaccinees’ group the predominat subset was T_N_**(B)**. The percentages of positivity for CD107a, the degranulation factor, within each phenotypic subpopulation in different study groups was assessed. This marker of cell activation was most prevalent within the T_CM_ cell subpopulation throughout all the study cohort **(C)**. The responses from non-stimulated cells were subtracted from the PPD responses. Three samples from each group were tested, and the means with corresponding ranges are shown for T_N_, T_CM_, T_EM_ and T_EMRA_ cell subsets.

CTLs from three samples of each group were analyzed on the basis of the following classification: CD45RA^+^CCR7^+^ naive (T_N_), CD45RA^–^CCR7^+^ central memory (T_CM_), CD45RA^–^CCR7^–^ effector memory (T_EM_), and CD45RA^+^CCR7^–^ terminally-differentiated effector memory (T_EMRA_) cells [20]. Representative dot plots are shown in Figure 4A. Figure 4B shows that the T_EM_ phenotype was predominant in the TB, LTBI, Surg. and Part. groups but not in the Mod. and the BCG groups (Figure 4B). As expected, in the BCG group the T_N_ was predominant, yet the other phenotypes with effector functions were detected as well. The degranulation marker CD107a proved more prominent in the T_CM_ than in the other phenotypic subpopulations. Together these results show that the phenotypic profiles of the cells in the circulation are similar in active TB, LTBI and in treated patients. The only group with a predominance of T_EMRA_ cells was the one with three-drug modern therapy, yet this difference needs to be confirmed in future experiments with larger study groups.

### Analysis of confounding factors

Significant negative correlation with age was found in CD4^+^IFN-γ^+^ T cells: *r* = -0.424, *p* < 0.01. Analysis of GrB and IFN-γ ELISPOTs resulted: *r* = -0.237, *p* = 0.053; *r* = -0.231, *p* = 0.060, respectively (Spearman’s rank correlation test). The gender had no effect on the results of immunological signatures when tested with the Mann-Whitney U-test.

## Discussion

We have earlier reported that effector memory CD4^+^ T cells persist in TB patients treated decades ago with different schemes, including bactericidal RMP [4]. Here, we present novel data demonstrating that functionally active antigen-specific CTLs expressing various effector molecules also persist in patients treated for TB. Indeed, the surface degranulation marker, i.e. CD107a, the cytotoxic signatures of CTLs, and their frequencies did not differ significantly between groups of treated patients and active TB or LTBI. Although the groups were small, GrB and IFN-γ-producing CTLs appeared to be more frequent in the Mod. than the Surg. group. Disappointingly, however, the functional and phenotypic profiling of CTLs performed with various methods and using different activation markers or combinations resulted in no discrimination between the active TB and the LTBI groups or any of the groups treated for TB.

Relatively small cohorts of patients with active TB and LTBI which were not matched for gender or age is a limitation of our study. Another limitation of ours as of many similar studies is our current inability to stratify TB infection into categories compatible with recently accepted notion that TB infection is in fact a continuum of infection severity starting from real latency through subclinical infection and ending with active disease [21]. From the point of view of the today’s knowledge the dichotomous division of infection into active TB and LTBI seems too simplistic.

The majority of antigen-specific CD8^+^ T cells were found to present with the T_EM_ and T_EMRA_ phenotypes, the finding consistent with previous studies [20,22]. T_CM_ and T_N_ cells were also found in the periphery of all the subjects. Some other studies have demonstrated antigen-specific naïve T cells (up to 90%) also in BCG vaccinated children and in TB patients [23,24], and T_CM_ cells (15%-25%) in TB patients at diagnosis and after four months of treatment [22]. The T_CM_ cell population also appeared to have the highest proportion of CD107a degranulation factor. This may indicate that this cell subset, while representing a minor population in the peripheral circulation could have an important role in inducing rapid immunologic protection against new challenges by the recently encountered pathogen.

It proved difficult to underpin a sustained correlation between the production of the cytolytic molecules, IFN-γ, and the expression of the CD107a. The expression, release and degranulation of these effector molecules as well as their presence in the granules appears highly dynamic and not interconnected [12,25,26], making this kind of an approach irreproducible. Therefore this approach does not seem clinically applicable to definitive differentiation of the various stages of TB infection. However, it may be intriguing in the future to reproduce cytotoxicity studies with other antigens, notably encoded by dormancy regulon (dosR) [27].

Although the function of CTLs has been studied both in human and murine TB models [28-33], their role in both infection defense and diagnostic potential remains unsettled. Compared to healthy TB contacts, patients with active TB presented with lower effector functions, e.g. GrB and Prf production [28,32]. When categorizing the patients with active TB into “mild” and “advanced” forms of infection (American Tuberculosis Society criteria) [30], the lytic activity of CTLs was found to be lower in the “advanced” than in the “mild” active TB or healthy PPD^+^ persons [30], suggesting exhaustion of CTLs. Exhaustion of T cells has been described also in many chronic viral infections, such as HIV, hepatitis C or hepatitis B virus [34,35]. It has been assumed that in chronic human viral infections, e.g. with cytomegalovirus (CMV) or herpes simplex virus 1 (HSV-1), the persistence of antigen will maintain the expansion of the functional memory T cell pool, the so-called inflationary T cells [36]. In a murine CMV infection model, inflated T cells in the spleen or blood were found functional and short-lived [37]. They can be renewed by cells primed during acute infection [37]. Here we also show that functional CD8^+^ T cells are present in the periphery in subjects adequately treated for TB decades ago. It thus appears that, like CMV, TB is a chronic infection exerting continued antigenic burden of T cells, finally leading to the expansion of antigen-specific T cell clones.

In CD8^+^ T cells subset, the cytotoxic potential and the expression of cytolytic molecules have been attributed especially to the CD45RA^+^CCR7^–^ T_EMRA_ phenotype, and to a lesser extent, to CD45RA^–^CCR7^–^ T_EM_ phenotype [38]. By contrast, the CD45RA^–^CCR7^+^ T_CM_ and CD45RA^+^CCR7^+^ T_N_ CD8^+^ T cells have been reported to express GrB after a 4-day priming [38]. In our study, functional T_EMRA_ and T_EM_ cells were found already after an overnight stimulation. In humans, GrB and Prf production and CD107a expression have been reported to correlate with cytotoxicity, in a study combining ELISPOT and CD107a FACS analysis with the results of Cr release assay [15-17]. By contrast, in a murine TB model, the CD107a expression has been correlated with cytotoxicity in primary, but not in secondary infection [39]. In another murine TB model [40], in secondary infection the cells contained Prf, but were not found cytotoxic. It has been speculated that T cells can degranulate and express CD107a even in the absence of the effector molecules [41]. In these studies [39-41], however, the reduction of CTL quality was caused by a lack of cytolytic molecule excretion.

The central dogma of TB pathogenesis has been that *Mtb*, an intracellular pathogen, infects and replicates, or sits quiescent in different cell lineages [6,42]. If the innate host immunity is inadequate to eradicate the invaded pathogen, the leading defense mechanism of adaptive immunity is a cell-mediated response, particularly of the Th1-type [6]. In response to the invasion, the host will “wall-off” the site of the infection by aggregating immune cells in a compact granuloma, a hallmark of TB. The granuloma is formed by various cell types among which CTLs constitute the majority. Interestingly, new discoveries obtained through imaging studies have enabled revisiting the role of granuloma in the containment of infection [43]. It is known that CTLs may be engaged to the target cells through the FAS ligand exertion, but this interaction may not necessarily eliminate the intracellular pathogen [43]. Thus, instead of killing, CTLs, like other immune cells, may aid bacterial proliferation.

Here we show that antigen-specific CTLs persist in TB patients treated with a three-drug regimen including bactericidal RMP. Where do these cells originate from, and how does the host benefit from maintaining these cell pools? One plausible but non-canonic explanation would be that during the over 9000-year evolutionary co-existence of *Mtb* with the host [44], a symbiotic relationship has evolved. The persistence of latency at some level would have up-regulated the basal activation state of the innate immunity against subsequent infections. The latency would have led to systemic activation of macrophages, and thus, through the polarized cytokine environment with a predominance of IFN-γ production, shifted the balance to the Th1 arm of the acquired immunity. In other words, the latency might have created a constant level of alertness against new invaders.

“The hygiene hypothesis” has been put forward to explain the growing rate of allergic conditions in the Western population [45,46]. This theory explains why the immune system will not be primed in infants living in environments sheltered from constant and excessive contact with bacteria, and later the immune response will be shifted towards the Th2 arm. The factors responsible for this priming are not clear: the role of bacteria belonging to the *Enterobactriacae* family has been discussed, while the possibility of the intracellular *Mtb* being beneficial has never been considered. Our observation that CTL subtypes persist after treated infection suggests that treatment may never lead to sterile immunity, but instead, once infected, a person would stay infected ever after. The fact that the LTBI is estimated to be present in almost 1/3 of the world’s population [47] might explain why, over the course of co-evolution, the LTBI might have turned even beneficial to the host. Of course, any potential benefit of latent infection for the survival of *homo sapiens* species requires that the disease mostly remains latent: indeed. An overt clinical TB will only develop in approximately 5% of those infected, while the rest of the cases remain latent throughout the individual’s lifetime [1]. We are fully aware that proposed hypothesis contradicts the established attitude towards LTBI as a harmful reservoir of TB. However, new evidence of latent infections protecting against other, more detrimental ones has been obtained with a murine model. It has been established that mice latently infected with intracellular Epstein-Barr virus (EBV) or CMV confer symbiotic protection against bacterial infection [48]. The protection is not considered complete, thus the hypothesis proposed does not contradict the fact that a re-infection can occur after a treated episode of TB.

## Conclusions

Functional CTLs persist in patients treated for TB even decades ago. On the basis on this finding it seems that the functional and phenotypical profiling of CTLs has a limited role as an adjunct immunodiagnostic tool for TB diagnostics. Further studies are warranted to learn about the clinical significance of persisting antigen-specific CTLs.

## Competing interests

The authors declare that they have no competing interests.

## Authors’ contributions

LS: Designed the study, performed the analyses, interpreted the data and collected clinical samples. PK: Collected clinical samples, interviewed the patients and analyzed demographic and clinical data. AK: Collected clinical samples and applied for the ethical clearances. HV, LP, RT: Collected clinical samples. BWW: Supervised the production of recombinant proteins. TT: Designed the study, applied for the ethical clearances, interpreted the data and with LS wrote the first draft. All authors contributed to the manuscript preparation, read, approved and accepted the final version.

## Pre-publication history

The pre-publication history for this paper can be accessed here:

http://www.biomedcentral.com/1471-2334/13/573/prepub
